# The Promise of Personalized TCR-Based Cellular Immunotherapy for Cancer Patients

**DOI:** 10.3389/fimmu.2021.701636

**Published:** 2021-07-30

**Authors:** Marion Arnaud, Sara Bobisse, Johanna Chiffelle, Alexandre Harari

**Affiliations:** ^1^Department of Oncology, Ludwig Institute for Cancer Research Lausanne, Lausanne University Hospital (CHUV) and University of Lausanne (UNIL), Lausanne, Switzerland; ^2^Center of Experimental Therapeutics, Department of Oncology, Lausanne University Hospital (CHUV), Lausanne, Switzerland

**Keywords:** cancer immunotherapy, T cell receptor, gene transfer therapy, adoptive cell transfer, cell engineering, T cells

## Abstract

Mutation-derived neoantigens are now established as attractive targets for cancer immunotherapy. The field of adoptive T cell transfer (ACT) therapy was significantly reshaped by tumor neoantigens and is now moving towards the genetic engineering of T cells with neoantigen-specific T cell receptors (TCRs). Yet, the identification of neoantigen-reactive TCRs remains challenging and the process needs to be adapted to clinical timelines. In addition, the state of recipient T cells for TCR transduction is critical and can affect TCR-ACT efficacy. Here we provide an overview of the main strategies for TCR-engineering, describe the selection and expansion of optimal carrier cells for TCR-ACT and discuss the next-generation methods for rapid identification of relevant TCR candidates for gene transfer therapy.

## Introduction

Pioneered by Rosenberg and colleagues, adoptive cell transfer (ACT) therapy is an immunotherapy strategy relying on the infusion of autologous tumor-infiltrating lymphocytes (TILs) to cancer patients. ACT demonstrated promising clinic outcomes in melanoma; with durable responses in 10-20% of patients (1, 2). Despite this progress, however, the majority of patients does not respond (3, 4) and the efficacy of ACT remains limited to melanoma and cervical cancer (5). One of the possible reasons for this limitation is the low frequency of antigen-specific T cells in TILs (1–3, 6–8). Furthermore, the proportion of bystander (i.e. tumor unrelated) TILs, such as viral-specific T cells, can be quite high in cellular products (9). Also, it was recently demonstrated that current cell culture conditions do not lead to a consistent clonal expansion of *ex vivo* TILs but rather lead to a biased immune repertoire (10). Altogether, these observations brought on the hypothesis that the proliferative potential of tumor antigen-reactive TILs is likely to be limited, therefore leading to their relative dilution *in vitro* by overgrowing bystander TILs.

**Figure 1:**
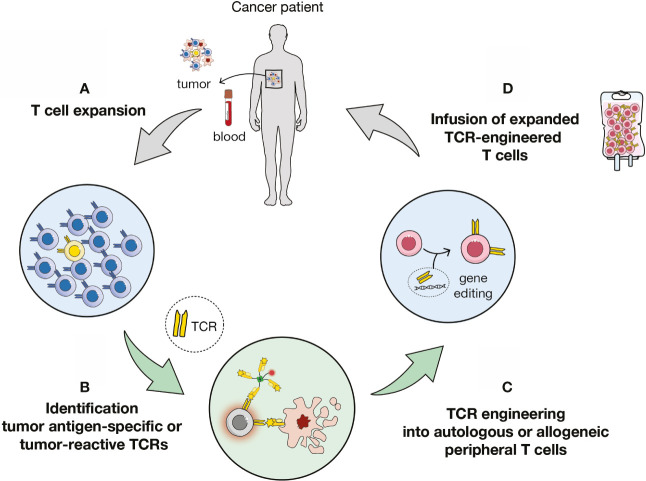
Overview of TCR-based immunotherapy. **(A)** Expansion of T cells from tumor or blood samples of patient or healthy donors. **(B)** Identification of antigen-specific or tumor-reactive T cells and of cognate T cell receptors (TCRs). **(C)** Transduction of autologous or allogenic carrier cells. **(D)** Adoptive cell transfer of TCR-engineered cells to the patient.

To circumvent these issues, the field is moving towards the genetic engineering of T cells to express either chimeric antigen receptors (CARs) (11) or tumor antigen-specific T cell receptors (TCRs) (**Figure 1**) (12–17). CD19-targeting CAR T cells mediated complete responses in about 80% of patients with B cell acute lymphoblastic leukemia cancer (18). Yet, CARs target tissue-restricted antigens expressed on the surface of tumor cells (19), thus limiting their applications (20), while TCR-transduced cells can target any surface or intracellular antigen. In this report, we will focus on TCR-based cellular immunotherapy (**Figure 1**).

TCRs are heterodimers consisting of disulfide-linked α and β chains, each with a variable and a constant domain (21, 22). The variable regions can bind the antigen-MHC complex. The binding domain is constructed based on the recombination of multiple gene segments, leading to the great diversity of the TCR repertoire, with potentially >10^15^ distinct αβ TCRs (23–25). TCR gene-transfer therapy targeting tumor-associated antigens (TAAs), such as MART-1 and NY-ESO-1, achieved clinical responses ranging from about 10 to 60% of patients from different malignancies (12, 14, 26, 27). Despite these promising clinical outcomes, efficacy remained limited and important toxicities occurred (13, 27, 28). The field was then rejuvenated by the perspective of using TCRs targeting private neoantigens, which have emerged as clinically relevant targets (29, 30). In this review, we will discuss several issues including the common tools used for cells transduction, the optimal cells to transduce for ACT and the acceleration of the identification of relevant tumor-specific TCRs for gene transfer therapy.

## Strategies for TCR-Engineering of T Cells

### Viral Vectors

**Figure 2:**
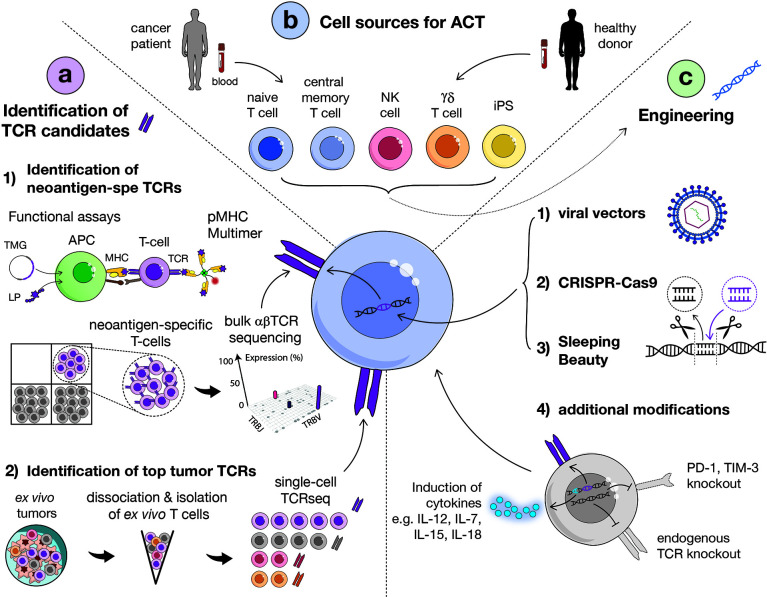
Overview of main strategies to identify relevant TCRs, methods for transduction and options of carrier cells. **(A)** Identification of TCR candidates *via* functional assays and sequential isolation of neoantigen-specific T cells based on pMHC multimer staining. Isolated neoantigen-reactive T cells can then be sequenced in bulk for the identification of dominant TCR clonotypes (1). Alternatively, tumor-specific TCRs can be identified directly from *ex vivo* fresh tumor, without intermediate culture, by sorting T cells by flow cytometry and performing single-cell TCR sequencing (2). (APC, antigen presenting cell; MHC, major histocompatibility complex; LP, long peptide; TMG, tandem minigene). **(B)** Several carrier cells can be used for TCR transduction, including αβ (CD4 or CD8) T cells, naïve or central memory cells, NK cells, γδ T cells and induced-pluripotent stem cells (iPSCs). Carrier cells can be derived from healthy donor or patient blood. **(C)** TCR engineering into carrier cells can be obtained *via* viral (1) or non-viral vectors [such as CRISPR-Cas9 (2) or Sleeping Beauty (3)]. Additional modifications of cells to enhance their functionality and *in vivo* persistency but also avoid TCR mispairing can be performed, such as the knock-out of endogenous TCR or immune inhibitor genes and the induction of cytokines (4).

As for CAR-T cell based therapy, viral vectors were widely exploited for *ex vivo* TCR gene transfer into recipient T cells (**Figure 2**). In clinical trials, the most common viral systems used are gamma retrovirus- (RV) and lentivirus-based vectors (LV), as reviewed in (31). Both RVs and LVs allow for stable integration and efficient long-term expression of exogenous TCRs. However, safety concerns to RVs and LVs remained, that are mainly insertional mutagenesis and neoplastic transformation (32), as well as generation of replication-competent viral particles. The latter limitations boosted the development of novel vector designs, such as addition of insulator sequences (33), disruption of the long terminal repeats for self-inactivating viral vectors (34, 35) or pseudotyping (36). Of note, RV transduction requires mitotic cells for the transgene to penetrate the nucleus and integrate in the genome and thus recipient cells must be activated beforehand. Conversely, LVs allow the effective transduction of a variety of not actively dividing and terminally differentiated cells. Yet, human resting T cells are scarcely susceptible to transduction by LVs and need to be minimally stimulated to enter the G1b phase (37, 38). Importantly. several studies highlighted a positive correlation between the proliferative potential of adoptively transferred cells and their *in vivo* persistency (1), sustaining the use of LVs in the clinic to limit recipient cell stimulation *in vitro*.

As alternative to RVs and LVs, adeno-associated virus-derived vectors (AAVs) can be used for TCR-engineering. AAVs are replication-deficient systems and are not known to induce any side effects, making them ideal candidates from a safety standpoint. AAV-based cancer treatments have not yet been used in the clinic, however multiple strategies have been developed and hold great promise for the future (39–41). It is to be noted that vector manufacturing remains time consuming (over six months) and expensive (42).

### Non-Viral Systems

Non-viral methods for stable gene transfer into T cells were investigated in CAR-T clinical trials and represent a future opportunity for TCR gene transfer clinical studies. Non-viral engineering strategies include transposon-based vector systems, such as “Sleeping Beauty”, by which a transgene of interest flanked by inverted terminal repeats is provided to target cells as discrete DNA molecule and randomly integrates in its genome when a transposase is co-supplemented *in trans* (i.e. mRNA, plasmid or protein) (43). Over the past decades, transposons and transposases have been extensively optimized to increase their activity and reduce toxicity (e.g. insertional mutagenesis) (44). Alternatively, exogenous TCRs can be transiently expressed *in trans* if transfected into T cells in the form of mRNA molecules or non-integrating vectors (45, 46). All these non-viral systems may be preferable to viral ones for the clinic because of their easier handling and cheaper production costs. Of important note, gene editing of T cells with TCRs, as opposed to CARs, has to face an extra challenge. Mispairing of exogenous and endogenous TCR chains can indeed occur and lead to off-target toxicity. Researchers have therefore developed several platforms to specifically silence the expression of the endogenous TCR, based on Sleeping Beauty, Zinc finger nucleases (47), transcription activator-like effector nucleases (TALEN) (48, 49), mega-nucleases or Clustered Regularly-Interspaced Short Palindromic Repeats (CRISPR)–Cas9 (50, 51) (**Figure 2**).

The CRISPR-Cas9 technology relies on short RNA sequences which are used to target the site of insertion instead of proteins and which are easily synthesized *in vitro*. As such, CRISPR-Cas9 enables the simultaneous targeting of multiple genome sites and site-specific mutagenesis, allowing the knockout (KO) of endogenous TCRs or of immune checkpoint such as PD-1 (52) and the induction of cytokine expression (e.g. IL-7, IL-12, IL-15, IL-18) (17) (**Figure 2**). The safety and feasibility of multiplex CRISPR-Cas9 gene editing of T cells was demonstrated in a study with advanced refractory cancer patients infused with autologous T cells KO for the three genes *TRAC*, *TRBC* and *PDC1* (PD-1 loci) and transduced with a NY-ESO1 TCR (53). Of interest, the KO by CRISPR-Cas9 of cytokine-induced SH2 protein (CISH), an immune checkpoint, was found to increase the *in vitro* proliferation and functionality of TCR-engineered T cells and such strategy will be further explored in an upcoming clinical trial (NCT04426669) (54). Despite technical challenges which still need to be overcome, in particular regarding the delivery systems currently based on AAV and transfection (55–59), CRISPR-Cas9 will likely become the method of choice for therapeutic gene engineering in the upcoming years.

## Selection and Expansion of Optimal Cells for ACT

### T Cells

T lymphocytes are the most common source of cells used as carrier for gene transfer therapy. Following TCR engineering into recipient cells, TCR-T cells need to be expanded to reach sufficient numbers for ACT. The primary starting material is most often autologous peripheral blood (**Figure 2**).

Thus far, TCR gene therapy mainly focused on CD8 T cells, which represent key players of ACT. In particular, a retrospective analysis of TIL ACT infusion products and clinical efficacy from 92 patients highlighted an association between the fraction of CD8 T cells and clinical benefit (60). There is, however, an emerging clinical relevance of CD4 T cells. Early evidence was provided by Tran and colleagues who showed the antitumor potential of neoantigen-specific CD4 TILs by ACT (61). Of interest, the direct cytolytic capacity of CD4 T cells was demonstrated (62–64). In addition, CD4 T cell’s help is essential to generate efficient tumor-reactive effector CD8 T cells (65), notably during the process of epitope spreading (29, 66). Yet TCR gene transfer therapy with CD4 tumor-specific TCRs is also limited by the challenging prediction and detection of MHC class II-restricted neoantigen-specific CD4 T cells, despite major advances (67, 68).

T cell differentiation state is critical for ACT. The profile of adoptively-transferred cells is indeed likely to affect their *in vivo* persistency and thus treatment efficacy. T cell differentiation states range from naïve to central memory, effector memory and finally terminally differentiated (EMRA) (69). Accumulating evidence shows that the effector phenotype acquired *in vitro* negatively impacts the antitumor potential of T cells *in vivo* (70), while ACT efficacy requires a long-term persistence of transferred cells. Thus, less-differentiated T cell populations that maintain self-renewal capabilities are preferred and were associated with improved clinical benefit (71, 72). Naïve and memory T cells maintain the highest proliferative potential combined to the most potent fitness and stemness (73–76) (**Figure 2**). Consistently, naïve and memory subsets were found more effective than effector T cells for ACT (70, 74–77). Furthermore, Hinrich and colleagues have demonstrated that naïve CD8 T cells had a higher anti-tumor potential for ACT as compared to central memory cells (77).

The T cell state is also modulated during the expansion phase *in vitro*. TCR-transduced T cells are commonly expanded with anti-CD3/CD28 beads in the presence of IL-2 (12, 38, 78). It has been demonstrated that the addition to the culture medium of alternative cytokines, such as IL-7, IL-15 and/or IL-21, enabled to the generation of less-differentiated TCR-engineered T cells thus leading to increased persistency and ultimately improved efficacy (71, 79–81).

Autologous T cells are available in limited quantities and their state is likely to be affected by the multiple rounds of cancer treatments which patients undergo before ACT, complicating the manufacturing process and the feasibility of TCR gene transfer therapy. Therefore allogenic universal T cells have also been exploited for gene transfer therapy (82) (**Figure 2**). A few issues are to be noted, including competition with endogenous TCRs, mispairing of TCR subunits, risk of off-target toxicity due to allogenic TCR-T cell infusion and ACT product rejection by the host (83). It is thus required to KO endogenous TCRs to improve the safety and efficacy of TCR-ACT with allogenic cells (49). For all these reasons, several alternative non-T cell types were evaluated.

### Alternative to Autologous αβ T Cells

Mensali and colleagues provided the first proof of concept that cells other than αβ T cells could be used as recipient cells for TCR gene therapy. They transferred a TCR into NK cell line, NK-92, and demonstrated efficacy *in vivo* (84). More recently, Parlar and coworkers have engineered NK cells with a tyrosinase-specific TCR and highlighted their cytolytic potential *in vitro* (85). A potential benefit of using NK cells as a carrier is their ability to remain cytotoxic in an MHC-independent manner. This could be of interest in case of MHC loss, which is a common immune suppressive mechanism exerted by tumors (86). Alternatively, γδ T cells can also be TCR-engineered (**Figure 2**), thus avoiding the issue of TCR mispairing. The latter strategy was proven efficient in leukemia (87). Furthermore, as T cell exhaustion is a critical component of ACT efficacy, there is an increasing interest in using induced-pluripotent stem cells (iPSCs) for gene transfer therapy (**Figure 2**). Nishimura and colleagues were able to generate iPSCs from antigen-specific CD8 T cells and to re-differentiate them. In this way, they obtained rejuvenated cells with longer telomeres and a high proliferative potential, making them fitter for therapy (88). The efficacy of TCR-engineered iPSCs was shown *in vivo* (89).

## Identification of Relevant TCRs for Gene Transfer Therapy

As mentioned previously, neoantigens are attractive targets for TCR-based therapy. To date, most neoantigens originated from non-synonymous mutations. T cell reactivity to neoantigens was associated with improved clinical benefit of immunotherapy, both immune checkpoint blockade (90–92) and ACT (93–95). Early reports showed promising results in terms of safety, feasibility and efficacy with neoantigen targeting immunotherapy either in the form of vaccination (29, 96–98) or ACT (30, 99). Strikingly, complete remissions were observed following infusion of neoantigen-reactive T cells, highlighting the potency of mutanome based therapies (30, 99).

The first key challenge lies in the identification of neoantigens, which is a long and tedious process and was reviewed elsewhere (100). Upon neoantigen identification, neoepitope-specific T cells are purified and their TCR is sequenced. Candidate TCRs are then cloned to validate their antigen specificity and tumor reactivity (**Figure 2**).

### Neoantigen Identification

Neoantigen-specific T cells, and hence their cognate TCR, can be identified in different samples including tumor or blood from patient or naïve T cells from healthy donors. Briefly, neoantigens can be identified from expanded TILs (7, 95, 101). However, due to low frequencies, sensitive detection sometimes requires antigen-specific *in vitro* stimulation (IVS). Of interest, we developed a novel strategy to improve the detection of neoantigens in TILs, based on the addition of pools of predicted neo-epitopes at the initiation of the TIL culture (7). Alternatively, TILs can be enriched prior to culture by sorting of dissociated tumor material *ex vivo*, based on various activation markers like PD-1 (CD279), OX40, CD137 (4-1BB), CD39 and CD103 (102–106).

Peripheral blood lymphocytes (PBLs) can also be exploited for neoantigen identification. IVS with antigen presenting cells (APC) loaded with neoantigen candidates have been extensively used to detect neoantigen reactivity (7, 30, 96, 107–110). Prior enrichment strategies have also been used by different groups including: the isolation of memory (107) or naïve T cells (108) or the sorting with PD-1 (109). Most identification processes use PBLs from autologous origin but it has been shown to be possible from allogenic sources as well (108).

### Neoantigen-Specific T Cell Isolation and TCR Repertoire Analysis

Upon their identification, neoantigen-specific T cells can be purified based on pMHC multimer staining or based on the up-regulation of activation markers following specific-activation, such as 4-1BB and OX40 (7, 107, 108, 111, 112). Isolated cells then undergo bulk α and β TCR sequencing in order to select the dominant α and β TCR clonotypes. Of note, TCR repertoire analysis strategies are challenging due to the high diversity of TCR repertoires (113). Advances in next-generation sequencing has improved the interpretation of TCR repertoires. Using RNA as a source of material allows allelic exclusion thereby avoiding to overestimate repertoires diversity. RNA is also more sensitive than DNA despite being less quantitative due to variation in expression levels (114). Among the different sequencing methods, multiplex polymerase chain reaction (PCR) (115) remains the most commonly used strategy, despite misrepresentation of clonotypes proportion introduced by heterogeneity in primers efficiency (116). Other approaches rely on the addition of adaptors prior to PCR amplification (117, 118), such as the 5’ RACE PCR or TCR amplification following gene capture (119). For each method, the bias in quantification reduces the ability for easy pairing of α and β chains, which is required for therapeutic applications. A concept based on multiple sequencing and combinatorial analysis was developed to pair αβ TCR chains, yet this strategy is limited to high-frequency clonotypes and requires large cell numbers (120). To avoid the above-mentioned limitations, both T cell cloning or single-cell sequencing can be used.

### Antigen Specificity Validation of Candidate TCR Pairs

Interrogation of neoantigen-specificity and antitumor-reactivity of candidate TCRs can be assessed following the expression of TCR candidates into recipient cells, a strategy hereafter referred as TCR cloning. Antigen-specificity can be challenged by transducing TCRs into activated PBLs or Jurkat T cells which are then co-cultured with APCs loaded with neoantigens (29, 86, 94, 95, 99, 101, 102, 107, 121–123). Next, tumor-reactivity can be measured by co-culture of TCR-engineered T cells with autologous tumor cells or APCs pulsed with tumor lysate (86, 94, 101, 102, 121, 124, 125). To enable a rapid identification of neoantigen-reactive TCRs, Paria and colleagues have developed a TCR cloning methodology using Jurkat T cells electroporated with RNAs encoding TCR α and β chain, respectively (126). The benefit of this approach lies in the use of RNA electroporation, which is faster and more efficient than TCR transduction by genetic engineering. TCR expression is transient but sufficient for TCR interrogation. Interestingly, they used the Jurkat luciferase system (under NFAT promotor) which is a rapid and easy read-out.

Of important note, the reactivity of validated neoantigen-specific TCRs to the wild-type peptide should be evaluated to avoid autoimmunity and thus ensure patient safety. This can done by performing a peptide dose response with APCs (86, 94, 95, 99, 102, 107). Another method consists of using a high-throughput genetic platform (127).

### Selection of Tumor-Specific TCRs By Single-Cell Technologies

As an alternative to the aforementioned time-consuming strategies (based on TCR isolation and downstream TCR validation), new developments enable the direct identification of tumor-specific TCRs by pre-selecting the most frequent clonotypes from fresh tumor samples (121) (**Figure 2**). Selected αβ TCR pairs are then challenged against tumor cells by TCR cloning. Others have however found that the tumor reactivity of intra-tumoral TCR repertoire was low (125), highlighting a potential limitation of using this strategy for gene transfer therapy. Identified tumor-reactive TCRs can then be examined retrospectively for antigen specificity using ligand discovery approaches, based for instance on trogocytosis, a process in which T cells exchange membrane proteins with APC presenting candidate antigens (128). Other possibilities for TCR ligand discovery include the use of chimeric signaling and antigen presenting bifunctional receptors (SABRs) (129). It is to be noted that the development of microfluidic-based platforms will accelerate and automate the isolation of relevant TCRs for TCR-ACT in the near future (130–132).

Instead of direct TCR identification, combined single-cell transcriptomics and TCR sequencing (133) could be exploited to define intra-tumoral signatures of neoantigen-specific or tumor-reactive TCRs (134). Old-generation single-cell methodologies are tedious and allow the sequencing of a limited number of cells (135, 136). Single-cell TCR sequencing and profiling of T cells was recently eased by the development of a commercially available strategy by 10x Genomics (137–139). Briefly, the latter microfluidic-based technology is able to generate an emulsion containing one trapped cell with a uniquely barcoded bead and, upon cell lysis polyadenylated mRNA is captured. The process results in barcoded libraries, which undergo both downstream single-cell TCR sequencing and transcriptomic profiling. Overall, 15’000-20’000 cells can be covered. The 10x Genomics strategy allows the analysis of clones in a timely manner and was applied for the identification of a signature of clone persistency in the circulation after ACT (140). In the near future, intra-tumoral signatures of neoantigen and/or tumor-reactive TCRs may be defined based on a comprehensive database of TCRs combined to their *ex vivo* transcriptomic profiling. We foresee that these signatures will enable the direct identification of relevant TCR pairs from *ex vivo* tumor, which will considerably facilitate and accelerate the selection of TCR candidates for gene transfer therapy.

## Discussion and Future Perspectives

In this review, we provided an overview of the available therapeutic engineering systems, but also of the optimal carrier cells for TCR gene transfer therapy, and finally we described strategies to identify new TCR candidates. The field is constantly evolving and future advances are likely to reshape the landscape of TCR-ACT. Multiple clinical trials are currently ongoing in different types of cancer (17) and the results are awaited with much anticipation. In the future, we could take advantage of unique intra-tumoral transcriptomic signatures from identified neoantigen-specific or tumor-reactive TCRs. These signatures may then be used to directly and rapidly fish out TCRs of interest for gene transfer therapy. Challenges of TCR-ACT remain, in particular immune-editing following therapy, ultimately leading to immune evasion and progression (86). To increase the efficacy of TCR-ACT therapy and counter potential immune-editing, multiple TCRs should be targeted, which is currently possible with the CRISPR-Cas9 technology. Future strategies may also focus on the simultaneous targeting of MHC-restricted epitopes together with membrane antigens. Importantly, to extend the reach of therapy and benefit more cancer patients, ‘shared’ neoantigens (141, 142) arising from ‘hotspots’ mutations shared between unrelated individuals should be preferentially targeted and these approaches will be further explored in upcoming clinical trials (e.g. NCT03190941). Additionally, it is likely that next-generation improvement of carrier cells will further potentiate TCR-ACT efficacy. Given the plethora of existing technologies and their constant amelioration, together with the many ongoing clinical trials, we expect the immune-oncology field to be fundamentally modified by TCR gene transfer therapy in the upcoming years.

## Author Contributions

All authors contributed to the article and approved the submitted version.

## Funding

This research was supported by the Ludwig Institute for Cancer Research and grant 310030_182384 from the Swiss National Science Foundation (A.H.).

## Conflict of Interest

The authors declare that the research was conducted in the absence of any commercial or financial relationships that could be construed as a potential conflict of interest.

## Publisher’s Note

All claims expressed in this article are solely those of the authors and do not necessarily represent those of their affiliated organizations, or those of the publisher, the editors and the reviewers. Any product that may be evaluated in this article, or claim that may be made by its manufacturer, is not guaranteed or endorsed by the publisher.
